# Transcriptome Profiling Reveals Disruption of Innate Immunity in Chronic Heavy Ethanol Consuming Female Rhesus Macaques

**DOI:** 10.1371/journal.pone.0159295

**Published:** 2016-07-18

**Authors:** Suhas Sureshchandra, Maham Rais, Cara Stull, Kathleen Grant, Ilhem Messaoudi

**Affiliations:** 1 Graduate Program in Genetics, Genomics and Bioinformatics, University of California Riverside, Riverside, California, United States of America; 2 Division of Biomedical Sciences, School of Medicine, University of California Riverside, Riverside, California, United States of America; 3 Division of Neurosciences, Oregon National Primate Research Center, Oregon Health and Science University, Beaverton, OR, United States of America; University of Kentucky, UNITED STATES

## Abstract

It is well established that heavy ethanol consumption interferes with the immune system and inflammatory processes, resulting in increased risk for infectious and chronic diseases. However, these processes have yet to be systematically studied in a dose and sex-dependent manner. In this study, we investigated the impact of chronic heavy ethanol consumption on gene expression using RNA-seq in peripheral blood mononuclear cells isolated from female rhesus macaques with daily consumption of 4% ethanol available 22hr/day for 12 months resulting in average ethanol consumption of 4.3 g/kg/day (considered heavy drinking). Differential gene expression analysis was performed using edgeR and gene enrichment analysis using MetaCore™. We identified 1106 differentially expressed genes, meeting the criterion of ≥ two-fold change and p-value ≤ 0.05 in expression (445 up- and 661 down-regulated). Pathway analysis of the 879 genes with characterized identifiers showed that the most enriched gene ontology processes were “response to wounding”, “blood coagulation”, “immune system process”, and “regulation of signaling”. Changes in gene expression were seen despite the lack of differences in the frequency of any major immune cell subtype between ethanol and controls, suggesting that heavy ethanol consumption modulates gene expression at the cellular level rather than altering the distribution of peripheral blood mononuclear cells. Collectively, these observations provide mechanisms to explain the higher incidence of infection, delay in wound healing, and increase in cardiovascular disease seen in subjects with Alcohol use disorder.

## Introduction

Current statistics shows 75% of adult men and 63% of adult women regularly drink alcohol. Alcohol Use Disorder (AUD), defined as the weekly use of ≥15 drinks for men and ≥8 drinks for women (1 drink equals 15 g of ethanol), is estimated to affect 9.4% of male and 4.7% of female adult US population [1]. AUD leads to decreased barrier function [2], liver damage [3], cardiovascular disease [4, 5], poor vaccine response and increased susceptibility to bacterial and viral infections resulting in overall increased mortality [6, 7]. Compared to men, women appear at a higher risk of developing ethanol-related diseases such as coronary heart diseases and stroke across all levels of consumption [8, 9]. Although some studies suggest that this increased susceptibility may be explained by the higher blood ethanol levels achieved after drinking [10], the mechanisms underlying this increased sensitivity are poorly understood.

Currently, there are only a handful of studies that have systematically examined sex differences in the effects of ethanol on the immune and inflammatory responses and most of these studies have been conducted in rodent models. Clinical studies aimed at the systematic exploration of dose-dependent, sex-specific immune regulation in response to chronic ethanol exposure pose significant challenges such as obtaining accurate information about the ethanol dose/exposure history, the presence of confounding factors such as smoking, the use of recreational or illicit drugs, and nutritional deficits. Additionally, individuals who suffer from AUD tend not to participate in research studies [11].

In this study, we leveraged a nonhuman primate model of voluntary self-administration to define the impact of chronic heavy ethanol consumption on immune homeostasis in young adult female macaques [12]. In this model, macaques are trained to self-administer ethanol first using a schedule-induced polydipsia to establish ethanol drinking, then allowing the monkeys access to both 4% ethanol and water for 22 h/day [13]. The animals segregate into categorically heavy and non-heavy drinkers and these patterns remain stable for greater than 12 months [14]. Using this model, we previously demonstrated that chronic ethanol consumption leads to reduced growth factor production by peripheral blood mononuclear cells due to changes in microRNA and transcription factor expression [15]. We also recently showed that robust changes in innate immune gene expression in animals that routinely drank to intoxication underlie defects in vaccine responses [16]. As a follow up, in the current study, we used peripheral blood mononuclear cells (PBMC) samples from 6 female macaques, which were categorized as heavy drinkers and 3 controls to define the impact of chronic heavy ethanol exposure on immune cell numbers and overall gene expression changes. Additionally, to identify changes at the protein level, we measured plasma levels of cytokines, chemokines and growth factors in both groups of animals.

Our analysis indicates that, although there was no difference in the numbers of circulating white blood cells between the controls and the drinkers, there were significant changes in gene expression. Specifically, genes involved in blood coagulation and the development of heart disease were up-regulated, while genes involved in innate immunity and inflammation were dysregulated. Furthermore, we report down-regulation of a number of transcription factors important in regulating inflammatory gene expression, including *STAT3*, the protein levels of which we previously reported was suppressed with ethanol consumption [15]. Additionally, expression patterns of several histone genes and chromatin modifiers were differentially regulated by heavy drinking suggesting a global regulation of gene expression changes in PBMC upon chronic exposure to ethanol.

## Results

### Chronic heavy ethanol consumption does not alter circulating immune cell frequency

The 6 ethanol-consuming monkeys were classified as heavy drinkers, based on their average daily ethanol intake and resultant blood ethanol concentration (BEC). Average individual BEC ranged from 41.2 (mg%) to 96.4 (mg%) while the average daily ethanol consumption ranged from 3.29 to 5.17 g/kg (Table 1), which translate into human equivalents of 11 to 18 drinks per day. Hematological analysis showed no differences in circulating white blood cells between the ethanol-consuming and control animals (S1A Fig). Moreover, flow cytometry analysis did not reveal any differences in frequency of circulating CD4 T, CD8 T, CD20 B cells, dendritic cells and monocytes (S1B Fig). In contrast, plasma levels of IL4, IL7, IL8, MIP1β, and SDF1α were significantly increased, while VEGFD levels were significantly decreased in heavy drinkers, indicative of changes in cellular function (S1C Fig).

**Table 1:** Summary of drinking behavior of animals used in this study.

**Animal ID**	**Weight Pre-EtOH (kg)**	**Weight at Necropsy (kg)**	**Mean BEC (mg%)**	**Mean daily intake (g/kg/day)**
**FC1**	4.46	6.80	0	0
**FC2**	4.63	5.80	0	0
**FC3**	4.36	4.85	0	0
**FH1**	4.16	4.95	49.8	3.93
**FH2**	4.64	6.30	41.2	3.29
**FH3**	4.56	5.20	74.9	4.89
**FH4**	4.34	4.60	60	4.02
**FH5**	4.69	5.55	66.3	3.94
**FH6**	4.03	4.85	96.4	5.17

### Chronic heavy ethanol consumption results in significant changes in gene expression

**Fig 1:**
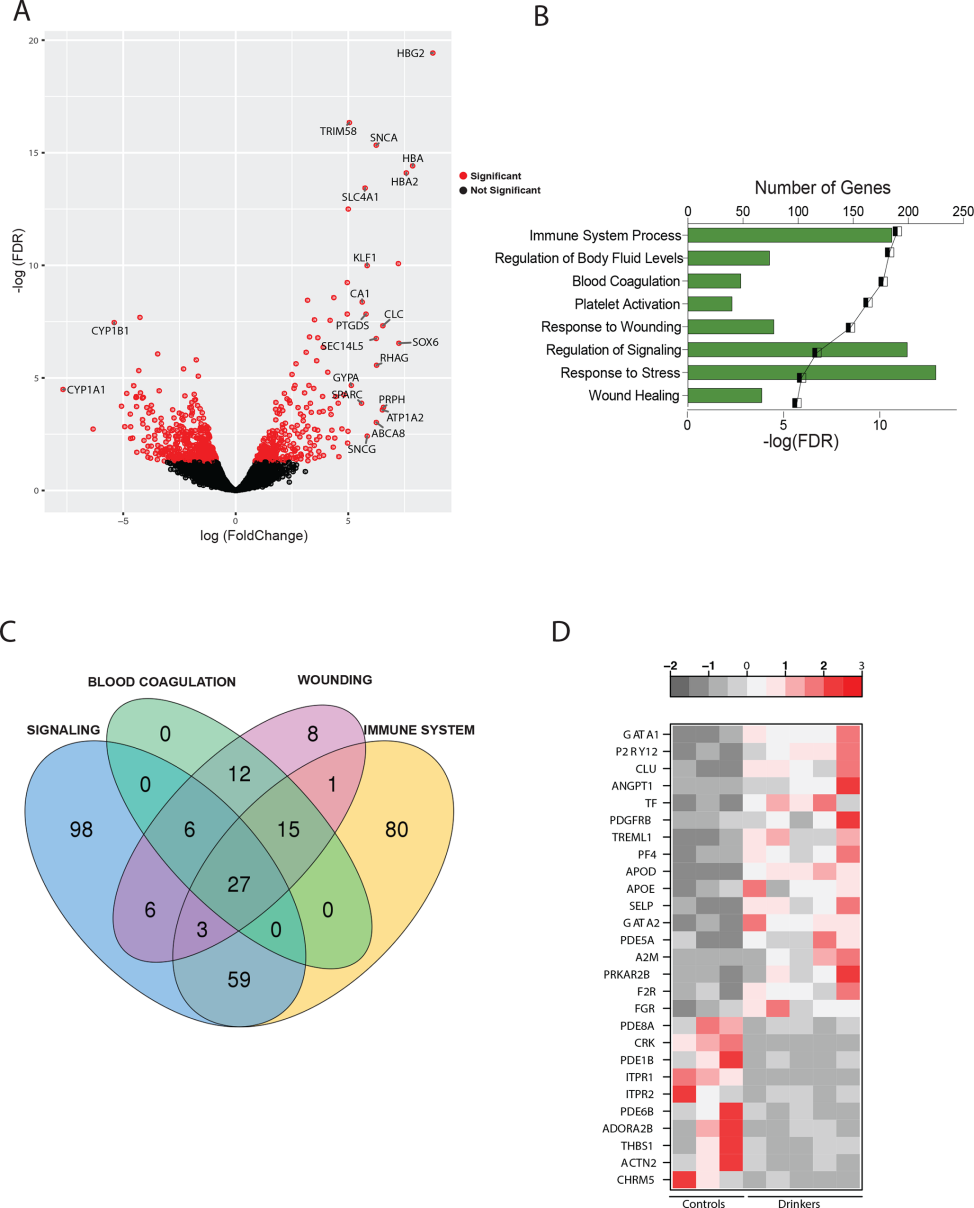
Chronic heavy ethanol consumption results in robust changes in gene expression within PBMC. (A) Volcano plot of global gene expression changes with red specks denoting genes with significant fold changes in gene expression, with gene names annotated for those with fold change ≥ 32. (B) Bar graph depicting the 8 most significant Gene Ontology (GO) terms enriched among all differentially expressed genes (DEGs), (C) Venn diagram depicting the overlap of genes enriched for four major GO terms—Signaling, Blood Coagulation, Wounding and Immune System Process. (D) Heatmap of the 27 differentially expressed that belong to all four GO processes—red depicts higher expression and grey, lower expression.

RNASeq was used to compare the PBMC transcriptomes between ethanol-consuming and ethanol-naïve animals. PBMC library from one of the ethanol-consuming animals failed, which was removed from all subsequent analyses. Principal component analysis (S2A Fig) clearly distinguishes the transcriptional profiles of ethanol naïve and ethanol-consuming animals. Comparing the transcriptomes of the 3 controls and 5 heavy drinkers resulted in 1106 differentially expressed genes (DEGs) with an FDR-corrected p-value of 0.05 and a fold change (FC) ≥ 2 (Fig 1B), with 445 up- and 661 down-regulated DEGs.

Some of the most highly up-regulated DEGs are genes involved in different stages of heme synthesis, notably *HBG2* (Hemoglobin γ2, FC = 433), *HBB* (β, FC = 332), *HBA* (α, FC = 232), *HBA2* (α2, FC = 191), *HBM* (μ, FC = 40) as well as *ALAS2* (5-aminolevulinate synthase, FC = 151) and *AHSP* (α hemoglobin stabilizing protein, FC = 19.6) (Fig 1A). The most highly repressed DEGs include *CYP1A1* and *CYP1B1* (Cytochrome P450 family 1 subfamily A and B polypeptide 1, FC = 203 and FC = 42.1 respectively), which are involved in the metabolism of toxins and carcinogens [17]; *AHRR* (Aryl hydrocarbon receptor repressor, FC = 37), which responds to xenobiotic stimulus [18]; and amino acid transporters such as *SLC7A11* (Solute carrier family 7 member 11, FC = 25.7) and *SLC24A1* (FC = 19) [19].

To understand the biological impact of these gene expression changes, we performed functional enrichment using MetaCore™. Since the software requires characterized gene identifiers for its analysis, this resulted in the removal of 227 DEGs. The remaining 879 DEGs enriched into several Gene Ontology (GO) processes notably ‘Immune System Process’, ‘Regulation of Signaling’, ‘Response to Wounding’ and ‘Blood Coagulation’ (Fig 1B). Since we observed a number of overlapping themes, we collapsed these terms into 4 major categories—Signaling (199 DEGs), Wounding (78 DEGs), Blood Coagulation (48 DEGs) and Immune System Process (185 DEGs).

A Venn diagram of the DEGs that mapped to these 4 GO terms revealed 27 common genes (Fig 1C). Of the 17 DEGs that were up-regulated (Fig 1D), several were involved in coagulation e.g. *P2RY12* (Purinergic Receptor P2Y, G-protein Coupled 12, FC = 14.8), which plays a critical role in maintaining thrombus stability [20]; *TREML1* (Triggering Receptor Expressed on Myeloid Cells like 1, FC = 9.1), which facilitates platelet aggregation [21]; and *PF4* (Platelet Factor 4, FC = 8.8), involved in sealing blood clots [22]. Other genes in this list include growth factors such as *ANGPT1* (Angiopoietin 1, FC = 12.4); adhesion proteins like *SELP* (P-Selectin, FC = 6.1) and *CLU* (Clusterin, FC = 12.5), and transcription factors *GATA1* (GATA binding protein 1, FC = 23.4) and *GATA2* (FC = 6).

**Fig 2:**
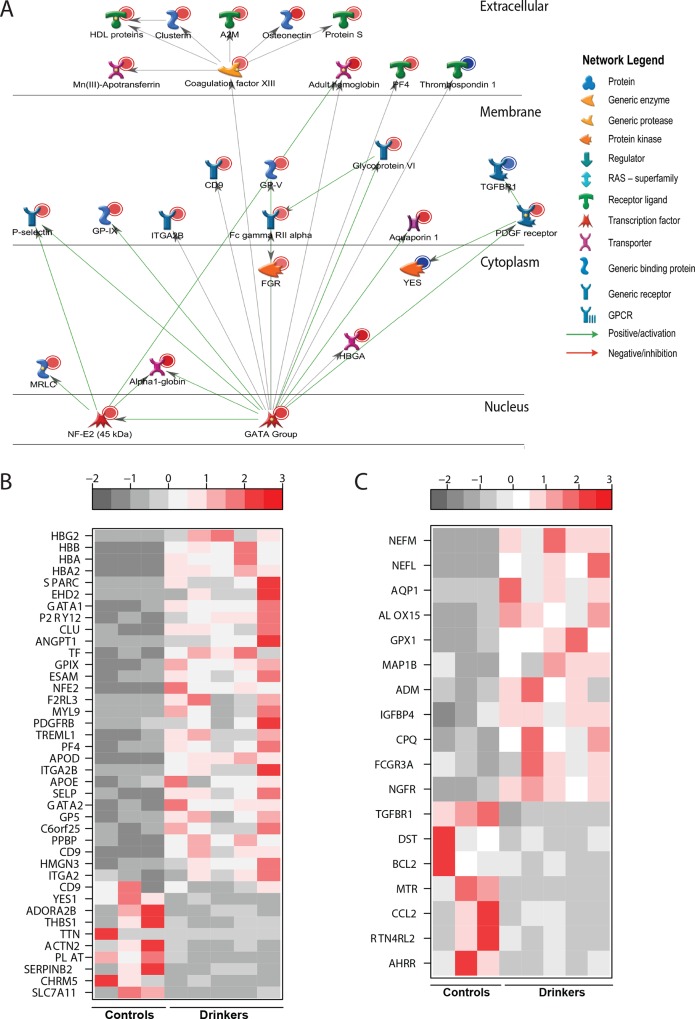
Chronic heavy ethanol consumption results in up-regulation of genes involved in blood coagulation and wound-healing. (A) Network of DEGs with direct interactions that mapped to “Wound healing”. (B) Heatmap of the 40 DEGs with a fold-change ≥ four-fold (30 up-regulated and 10 down-regulated) involved in “Blood Coagulation”. (C) Heatmap of the 18 DEGs that mapped to “Wound healing” but did not map to “Blood Coagulation”.

Several of the 10 common DEGs that were down-regulated (Fig 2B) play crucial roles in signaling notably *ADORA2B* (Adenosine A2b Receptor, FC = 4.4), which regulates adenosine levels during T cell activation [23]; phosphodiesterases such as Phosphodiesterase 1B (*PDE1B*, FC = 2.2), *PDE6B* (FC = 3.3) and *PDE8A* (FC = 2); and *THBS1* (Thrombospondin 1, FC = 4.9), which is critical for resolution of inflammation [24].

### Chronic heavy ethanol consumption results in up-regulation of genes involved in blood coagulation but decreases expression of genes critical for wound healing

The 48 DEGs that mapped to GO terms associated with coagulation also mapped to GO terms associated with wound repair. Overall, we noted an up-regulation of pro-clotting genes and down-regulation of anti-clotting genes (Fig 2A and 2B). Specifically, hemoglobin genes *HBG2* (FC = 433), *HBB* (FC = 332), *HBA2* (FC = 191), hemoglobin-stabilizing protein *AHSP* (FC = 19.6) and *NFE2* (Nuclear Erythroid Factor 2, FC = 10.5) were highly up-regulated. In addition, coagulation factors such as *F13A1* (Coagulation Factor XIII, FC = 3.4) and *F2R* (Coagulation Factor II receptor, FC = 2.8) were over-expressed. Moreover, we observed up-regulation of erythropoietic transcription factors *GATA1* (FC = 23.4) and *GATA2* (FC = 6), which regulate the switch from fetal to adult hemoglobin and activate the development of hematopoietic cell lineages [25]. Finally, expression of several platelet adhesion molecules also increased notably: *SELP* (P-Selectin, FC = 6.1) [26, 27], up-regulated in response to vascular injury; *GP5* (Glycoprotein 5, FC = 5.9), which promotes platelet aggregation and adhesion to the site of vascular injury [28, 29]; and *ITGA2* and *ITGA2B* (Integrin Alpha 2 and 2B, FC = 4.2 and FC = 7.4, respectively), which mediate adhesion of platelets and immune cells to the extracellular matrix [30]. In contrast, positive regulators of fibrinolysis were down-regulated such as the potent thrombin inhibitor *SERPINB2* (Serpin Peptidase Inhibitor, Clade B member 2, FC = 7), and the plasminogen activator *PLAT* (Plasminogen activator tissue, FC = 2.7). Additionally, we observed increased expression of *SPARC* (FC = 48) and *SPARCL1 (*SPARC-like 1, FC = 19.3), which increase the production of matrix metalloproteinases and expression of growth factors at the site of injury [31].

The list of DEGs that mapped to wound healing but not blood coagulation is summarized in Fig 2C. The down-regulated genes include the monocyte chemoattractant *CCL2* (Chemokine Ligand 2, FC = 11.8) [32] and the angiogenic factor *TGFBR1* (Transforming growth factor receptors Beta 1, FC = 2.1) [33]. The up-regulated DEGs include several genes important for wound healing, notably *AQP1* (Aquaporin 1, FC = 24), which promotes cell migration during wound healing events was increased [34]. *ADM* (Adrenomedullin, FC = 4.9), which promotes angiogenesis and remodeling; and *GPX1* (Glutathione Peroxidase 1, FC = 10.8), an antioxidative enzyme that regulates wound healing [35].

### Chronic heavy ethanol consumption results in dysregulation of genes involved in innate immunity and immune system development

**Fig 3:**
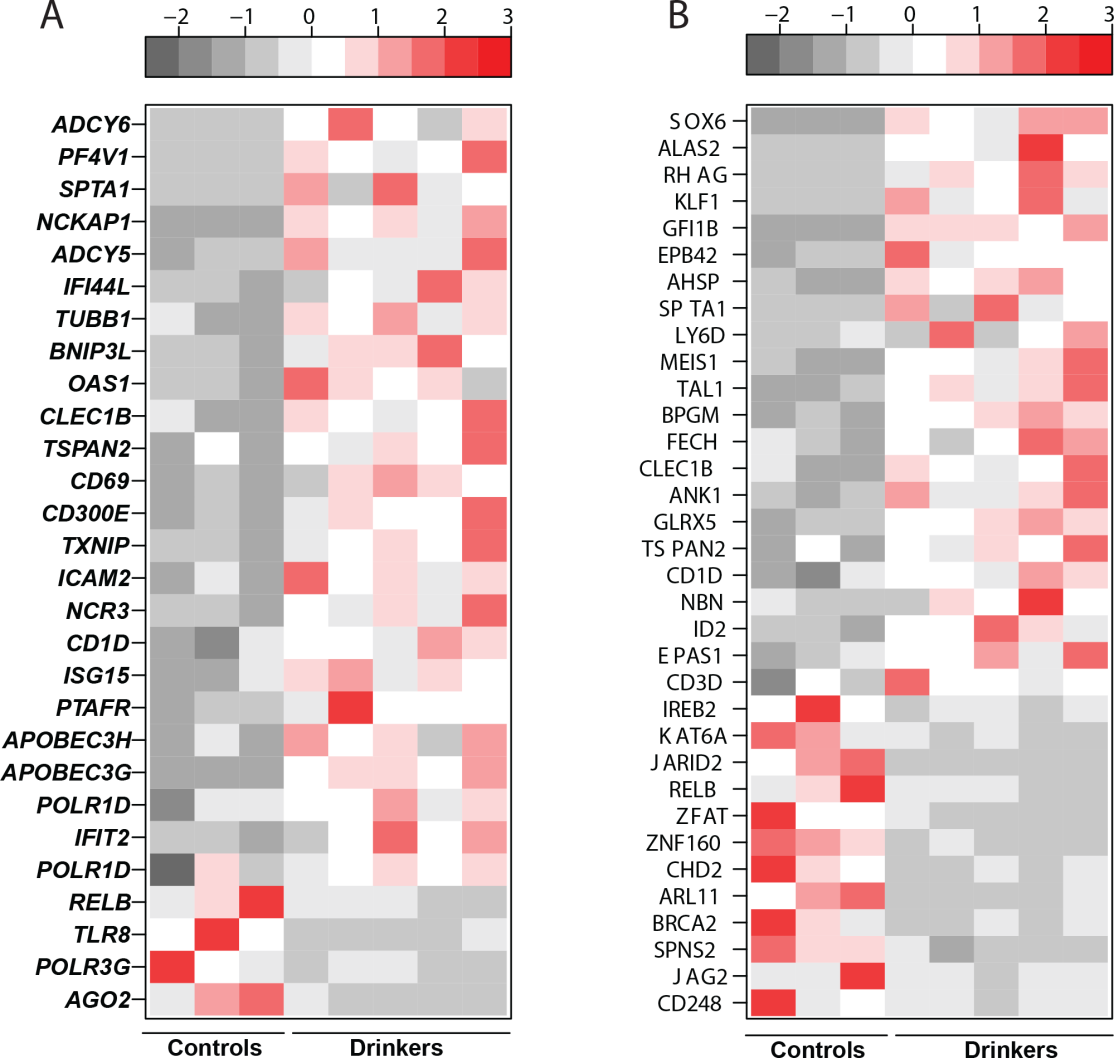
Chronic heavy ethanol consumption results in dysregulation of genes involved in innate immunity and immune system development. (A) Heatmap of DEGs that map to “Defense Response/Innate Immune Response” (B) Heatmap of DEGs that map to “Immune System Development”/“Myeloid Cell differentiation”.

Of the 185 DEGs that mapped to Immune System Process, 46 DEGs also mapped to “Wound Healing” and 89 to “Signaling”. Additional bioinformatics analysis of the expression profiles of the 185 genes that mapped to ‘Immune System Process’ using the Immunological Genome Consortium database indicated that a significant number of these genes are highly expressed in innate immune cells–monocytes and dendritic cells (DCs). These results suggest that ethanol consumption exerts the biggest transcriptional impact on the innate immune system (S3A Fig). Indeed, the 80 DEGs that mapped exclusively to “Immune System Process” further enriched into GO processes: Defense Response/Innate Immune Response (28 DEGs) (Fig 3A), and Hematopoiesis/Immune System Development/Myeloid Cell Differentiation (34 DEGs) (Fig 3B).

Genes involved in defense response/innate immune response were largely up-regulated (Fig 3A). This list includes several interferon-induced genes including *IFI44L* (Interferon-Induced Protein 44 Like, FC = 5.7), *IFIT2* (Interferon-Induced Proteins with Tetratricopeptide repeats 1B, FC = 2.2), as well as *ISG15* (ISG15 Ubiquitin-Like Modifier, FC = 2.7). In addition, innate immune genes known for their role in antiviral defense, such as viral dsRNA sensor *OAS1* (2’-5’ Oligoadenylate Synthetase 1, FC = 4.3) [36]; RNA editing enzymes *APOBEC3G* (FC = 2.3) and *APOBEC3H* (FC = 2.3) [37] were also up-regulated. Several receptors involved in host defense and cell activation were also up-regulated. This list includes *CD300E* (FC = 3.4), a receptor capable of activating innate immune responses in myeloid cells [38]; *CD1D* (FC = 2.8), a glycoprotein molecule responsible for selection of NKT cells [39]; and the adhesion molecule *ICAM2* (FC = 2.9). Furthermore, several T and NK cell activation markers were over-expressed, notably *CLEC1B* (C-Type Lectin Domain Family 1, Member B, FC = 3.7); *CD69* (FC = 3.4) [40, 41]; and, *TSPAN2* (Tetraspanin 2, FC = 3.4) [42] (Fig 3A).

Some of the defense response genes that were under-expressed include viral single-stranded RNA sensor *TLR8* (Toll Like Receptor 8, FC = 2.2) [43]; *RELB* (V-Rel Avian Reticuloendotheliosus Viral Oncogene Homolog B, FC = 2.2), a subunit of the NF-kB complex with critical role regulating the transition from innate to adaptive immunity [44], and *AGO2* (Argonaute RISC Catalytic Component 2, FC = 3.3), an essential RNA binding protein that mediates remodeling of the microRNA repertoire [45].

Genes that mapped to “Hematopoiesis/Immune System Development/Myeloid Cell Differentiation” were also largely up-regulated. Most of these DEGs were primarily involved in erythroid function (Fig 3B), notably *ALAS2* (Erythroid-specific delta aminolevulinate synthase 2, FC = 151), which catalyzes the first reaction in the heme biosynthetic pathway [46]; *AHSP* (Alpha Hemoglobin Stabilizing Protein, FC = 19.6), which plays a role in stabilizing free alpha hemoglobin [47]; and *SPTA1* (Spectrin Alpha, FC = 18.4), responsible for maintaining the stability and flexibility of erythrocytes. A number of transcription factors involved in erythrocyte development were also up-regulated such as *SOX6* (Sex Determining Region Y—box 6, FC = 152), *KLF1* (Krueppel Like Factor 1, FC = 57.3), *MEIS1* (MEIS Homeobox 1, FC = 9.2), *GFI1B* (Growth Factor Independent Transcriptional Repressor 1B, FC = 31.5), and *EPB42* (Erythroid Membrane Protein Band 4.2, FC = 31) [48–52]. Among the genes down-regulated in this GO term were transcriptional repressors of immune system such as *ZFAT* (Zinc Finger Protein 406, FC = 2.4) [53, 54]; *BRCA2* (Breast Cancer 2, Early Onset, FC = 3.2) [55], and *ZNF160* (Zinc Finger Protein 160, FC = 2.5) [56].

### Heavy ethanol consumption dysregulates genes involved in innate immune signaling

**Fig 4:**
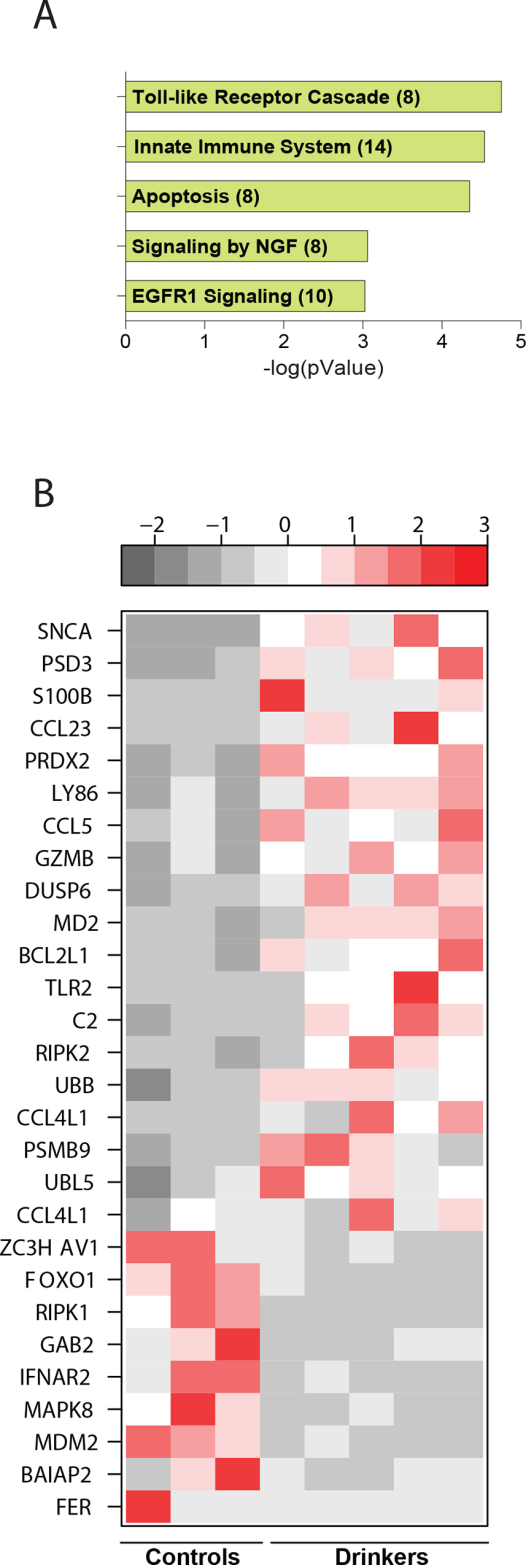
**Chronic heavy ethanol consumption results in dysregulation of genes involved in innate immune signaling** (A) Bar graph of 5 significant terms enriched among 89 genes mapping to “Immune System Process” and “Signaling” as predicted by InnateDB with the number of genes mapping to each term included in parenthesis (B) Heatmap of DEGs that map to “Innate Immune System” and “Apoptosis”.

Additional enrichment analysis was performed on the 89 DEGs that mapped to both ‘Immune System Process’ and ‘Signaling’ using InnateDB [57] allowing us to identify enriched signaling pathways from a smaller pool of genes in an unbiased way. These 89 genes mapped to ‘Innate Immune System’ and ‘Apoptosis’ (Fig 4A and 4B). Specifically, we observed up-regulation of a number of genes encoding inflammatory molecules such as *CCL23* (Chemokine C-C motif ligand 23, FC = 9.7), *CCL4L1* (Chemokine C-C motif ligand 4-like, FC = 2.3) and *CCL5* (FC = 3.7), which are all highly chemotactic for macrophage and NK-cell migration [58, 59]. Additional up-regulated genes of interest play a critical role in innate immune signaling, such as *TLR2* (Toll-like Receptor 2, FC = 2.7); *LY86* (Lymphocyte Antigen 86, FC = 4.1); *MD2* (Lymphocyte Antigen 96, FC = 3.2); and *RIPK2* (Receptor Interacting Protein Kinase 2, FC = 2.4), critical for NOD mediated activation of NF-κB and pro-inflammatory cytokine production [60].

Interestingly, a number of genes belonging to the Ubiquitin proteosome pathway were dysregulated in heavy drinkers. *UBB* (Ubiquitin B, FC = 2.3), involved in targeting proteins for proteolytic degradation by the proteasome [61]; *UBL5* (Ubiquitin-like protein 5, FC = 2.1) and *PSMB9* (Proteasome Subunit Beta 9, FC = 2.2) were up-regulated, whereas *MDM2* (Murine double Minute -2, FC = 2.5), a ubiquitin ligase [62], was down-regulated.

Furthermore, a number of genes with roles in regulating innate anti-viral immunity were dysregulated. For example, *PRDX2* (Peroxiredoxin, FC = 5.3), an anti-oxidant enzyme that contributes to antiviral activity of T-cells was up-regulated, whereas *ZC3HAV1* (Zinc Finger CCCH-Type, Antiviral 1, FC = 2), an anti-viral protein that mediates degradation of viral RNA [63] was down-regulated (Fig 4B).

Among the 98 genes that were exclusive to the GO term ‘Signaling’, InnateDB’s Network Analyst application revealed enriched STAT3 signaling (S4A Fig). *STAT3* (Signal Transducer and activator of transcription 3, FC = 2.1), which activates gene transcription of pro-inflammatory molecules via NF-KB activation pathway was down-regulated in heavy drinkers. Interestingly, we observed mixed changes in gene expression of its targets. Targets that play a role in inflammation and immunity such as *IL1R1* (Interleukin 1 Receptor, Type 1, FC =), *CCL2* (Chemokine C-C Motif Ligand 2, FC =) and *THBS1* (Thrombospondin 1, FC) were down-regulated. In contrast, targets that play a role in interferon signaling were all up-regulated such as *OAS1* (2’-5’ Oligoadenylate Synthase 1, FC = 4.3), *IFIT1* (Interferon-Induced Protein With Tetratricopeptide Repeats 1, FC = 5.9), *IFIT1B* (FC = 89), *IFI44* (Interferon-Induced Protein 44, FC = 5.4), and *IFI44L* (FC = 5.7).

### Chronic heavy ethanol consumption changes the expression of genes involved in heart diseases and cancer

**Fig 5:**
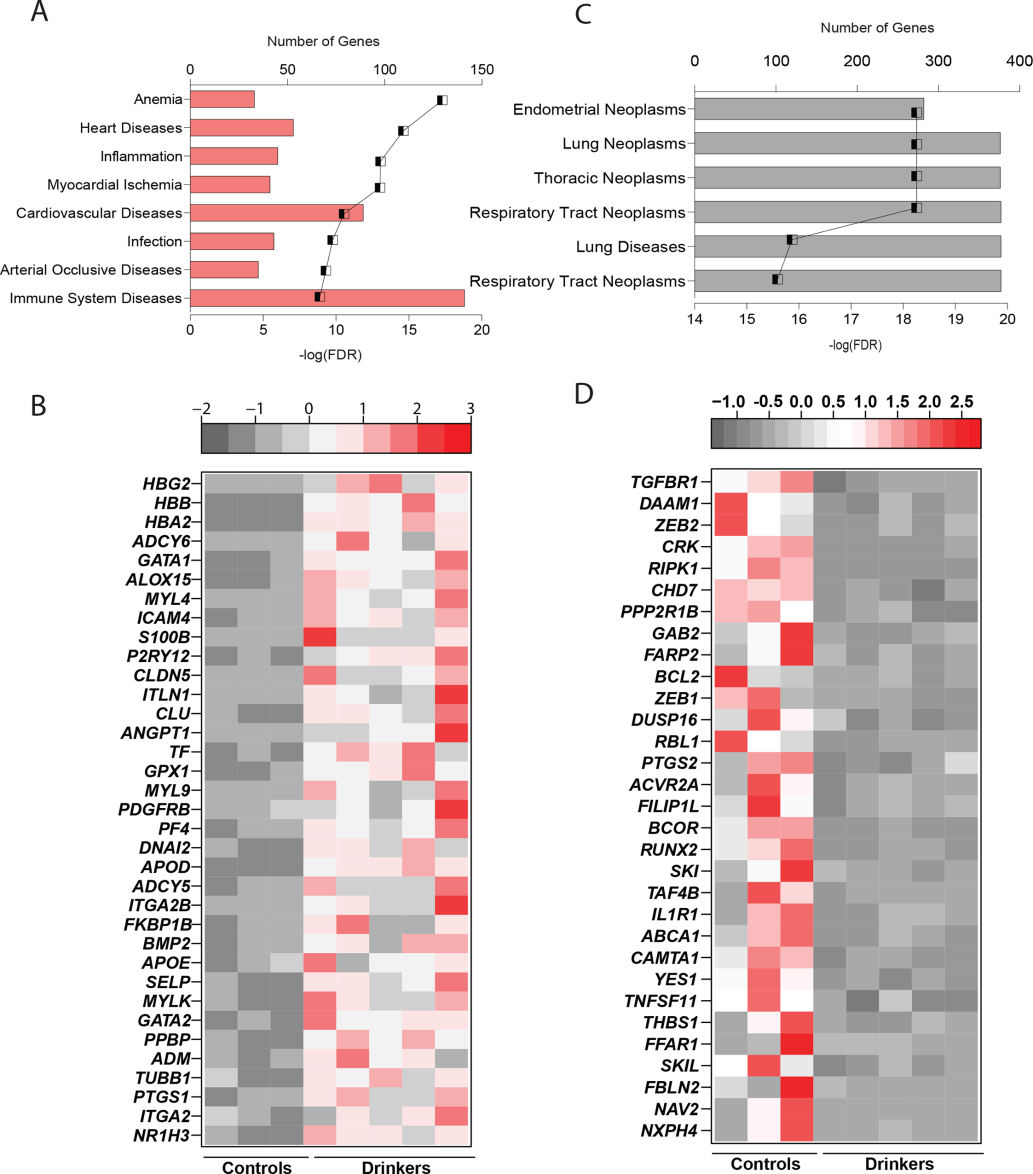
Chronic heavy ethanol consumption changes the expression of genes involved in heart diseases and cancer. (A) Bar graph depicting 8 disease terms enriched among the up-regulated genes. The line graph in both figures represents negative log (FDR) of the enriched term. (B) Heatmap of up-regulated genes involved in cardiovascular diseases. (C) Bar graph depicting 8 disease terms enriched among the down-regulated genes. (D) Heatmap of the down-regulated genes involved in cancer.

To get a better understanding of the biological impact of the gene expression changes, we conducted disease enrichment analysis for up- and down-regulated genes separately. Up-regulated DEGs primarily mapped to Anemia, Myocardial ischemia, and heart diseases (Fig 5A). Indeed, several of the up-regulated DEGs have documented association with cardiovascular risk when expressed at high levels (Fig 5B). This includes enzymes like *ALOX15* (Arachidonate-15-lipoxygenase, FC = 18.8) [64], *GPX1* (Glutathione Peroxidase 1, FC = 10.8) [65], adhesion molecule *SELP* (P-Selectin, FC = 6.1) [27], and the apolipoprotein *APOE* (FC = 6.3) [66].

Down-regulated genes enriched to several categories of cancer (Fig 5C). The 157 DEGs shared amongst these cancer terms contained transcription regulators such as *CAMTA1* (Calmodulin Binding Transcription Activator 1, FC = 4) [67], *RUNX2* (Runt-related transcription factor, FC = 3.5) [68] and *ZEB1* (Zinc Finger E-Box Binding Homeobox 1, FC = 2.6); and proto-oncogenes like *YES1* (FC = 4.4) [69] and *SKI* (FC = 3.6) [70](Fig 5D). Additionally, a number of signaling receptors also mapped to these GO terms including *FFAR1* (Free fatty acid receptor 1, FC = 5.1) [71], *TGFBR1* (Transforming Growth Factor, Beta Receptor 1, FC = 2) and *TNFSF11* (Tumor necrosis factor receptor superfamily 11, FC = 4.6) [72]. Interestingly, several zinc finger genes that regulate cell cycle progression like *ZC3H12C* (FC = 6.2) [73], were also down-regulated in heavy drinkers.

### Chronic heavy ethanol consumption results in changes in expression of epigenetic regulators

Our analysis revealed that a number of microRNAs were down-regulated in response to chronic ethanol consumption (Table 2). Of note, miR-27a (FC = 6.2), which regulates M2 macrophage polarization of human monocytes by targeting *IL10* [74, 75], was down-regulated. Importantly, three of its targets were up-regulated in our analysis: *PAQR9* (FC = 439) [76], *NR2F6* (Nuclear Receptor Subfamily 2, Group F, Member 6, FC = 3.1) [77] and *GATA2* (Phospholipase C-Like 2, FC = 2.3). Similarly, miR-24 (FC = 7.2) was under-expressed while several of its predicted targets were up-regulated: *NEFM* (Neurofilament Medium Polypeptide, FC = 345), *BNIP3L* (BCL2 Interacting Protein 3 Like, FC = 4.4). Finally, predicted targets of down-regulated miR-23a (FC = 6.7) such as *CA2* (Carbonic anhydrase 2, FC = 6.2) and *PTP4A2* (Protein Tyrosine Phosphate Type 4A, FC = 2.2) were also up-regulated. Interestingly, miR-23~27~24 clusters have been shown to regulate T-cell mediated immune homeostasis [78].

**Table 2:** Summary of down-regulated microRNAs and their up-regulated targets in drinkers.

**miRNA down-regulated in heavy drinkers**	**Fold Change**	**Gene targets up-regulated in heavy drinkers**
miR-23a	6.7	*PTP4A2*, *CA2*, *NCKAP1*, *XRCC2*, *GRTP1*, *MAP1B*
miR-24	7.2	*NEFM*, *BNIP3L*, *STRADB*, *EHD2*, *WWRT1*, *XRCC2*, *AMOTL2*, *FKBP1B*, *HBQ1*, *C6orf25*, *GSTO2*
miR-27a	6.2	*PAQR9*, *NR2F6*, *RGS2*, *RNF152*, *AMOTL2*, *GATA2*, *MAP1B*
miR-663	18.7	*MYL9*, *NRGN*, *CCTN*

**Fig 6:**
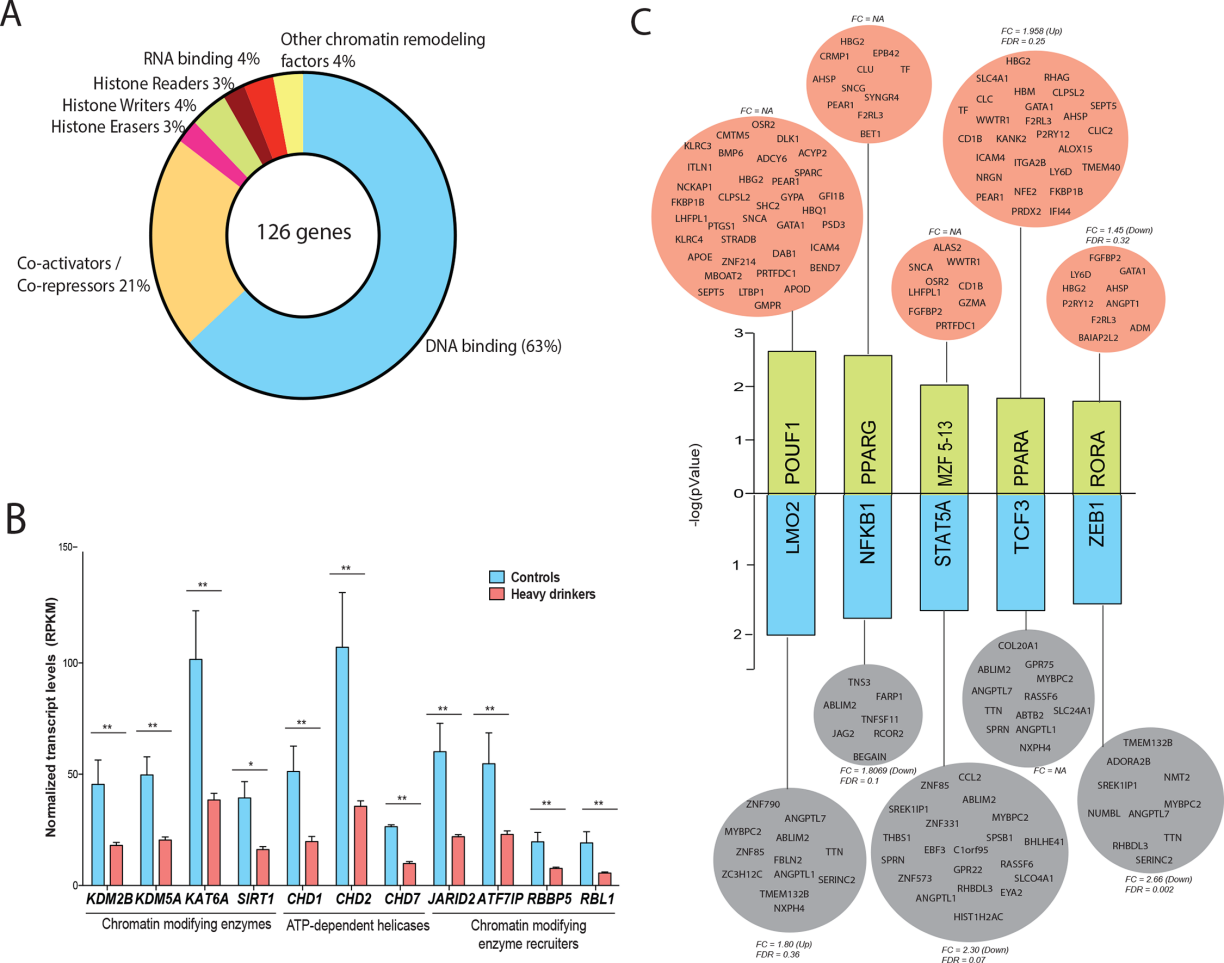
Chronic heavy ethanol consumption results in changes in expression of epigenetic regulators. (A) Functional profiles of the 128 down-regulated genes mapping to ‘Regulation of Gene Expression’ (B) Bar graph of expression levels (RPKM) of genes involved in chromatin remodeling (**—FDR of 5% and *—FDR of 10%). (C) Bar graph of 5 most significantly up- and down-regulated transcription factor networks. Green bars indicate up-regulated network and blue bars indicate down-regulated networks. Each bar is linked to a group of target genes (orange–up-regulated and grey down-regulated) that are differentially expressed in heavy drinkers.

Initial gene enrichment analysis of all DEGs using MetaCore showed GO processes ‘Regulation of Transcription’ and ‘Regulation of Gene Expression’ as significantly down-regulated in heavy drinkers (S2C Fig). A significant number of these 128 genes were involved in chromatin reorganization, particularly histone erasers (*HDAC9* (Histone deacetylase 9, FC = 2.8), histone writers *PRDM8* (PR Domain Containing 8, FC = 2.8) and histone readers (*CHD1* (Chromodomain Helicase DNA Binding Protein 1, FC = 2.1), and *CHD2* (FC = 2.5)) (Fig 6A and 6B). Interestingly, these enzymes catalyze post-translational modifications primarily on histone H3 [79]. Furthermore, coregulators of these enzymes such as *RBL1* (Retinoblastoma Like 1, FC = 2.9) were also down-regulated (Fig 6B). Additionally, a number of transcription factors that regulate cell proliferation, differentiation and transformation were down-regulated such as *AFF3* (Lymphoid Nuclear Protein4, FC = 2.7), *STAT3* (Signal Transducer and Activator of Transcription 3, FC = 2.1), *HIVEP1* (Human Immunodeficiency Virus Type I Enhancer Binding Protein 1, FC = 2.3), *FOXO1* (Forkhead Box O1, FC = 2.0), and *FOSB* (FBJ Murine Osteosarcoma Viral Oncogene Homolog B, FC = 2.3) [80–83].

Among the up-regulated transcription factors were *MEIS1* (Meis Homeobox 1, FC = 9.2), which regulates hematopoeisis and megakaryocyte lineage development [84]; *EPAS1* (Endothelial PAS Domain Protein 1, FC = 2.5), which regulates expression of oxygen-related genes [85]; *NFE2* (Nuclear Factor Erythroid 2, FC = 10.5), which regulates immune functions, redox homeostasis and intracellular signaling in dendritic cells [86]; and, *TXNIP* (Thioredoxin Interacting Protein, FC = 3), an oxidative stress mediator required for the maturation of NK cells [87].

To identify dysregulated transcription factor networks, we performed transcription factor binding site (TFBS) analysis of all 1106 genes using InnateDB (Fig 6C). The results from this analysis indicate up-regulation of transcriptional networks regulated by POUF1, an activator of hormone genes [88]; PPARG (Peroxisome proliferator-activated receptor gamma) and PPARA, critical regulators of inflammatory response [89, 90]; MZF5-13 (Myeloid Zinc Finger 5–13), which plays a crucial role in negative regulation of proliferation of hematopoietic progenitors [91] and RORA (Retinoic-Acid-Receptor-Related Orphan Nuclear Receptor Alpha), a nuclear receptor required for natural helper cell development in all tissues[92].

The down-regulated transcriptional networks were regulated by NFKB1 (Nuclear Factor of Kappa Light Polypeptide Gene Enchancer in B-cells 1), a subunit of NFKB, whose activation has been shown to be inhibited following acute ethanol exposure in vitro resulting in reduced production of inflammatory cytokines [93, 94]. Additional suppressed transcriptional networks include STAT5A (Signal Transducer and Activator of Transcription 5A) [95]; TCF3 (Transcription Factor 3) and LMO2 (T-cell translocation protein 2), which are essential regulators of hematopoiesis and lymphopoiesis [96, 97]; and ZEB1 (Zinc Finger E-Box Binding Homeobox 1) and TCF8, which play a key role in cellular differentiation [98].

## Discussion

The gender-specific impacts of alcohol consumption, including AUD are under-studied. In this study, we used RNA-Seq to characterize differences in gene expression in PBMC collected from female rhesus macaques after 12 months of chronic ethanol consumption using a model of voluntary ethanol self-administration that robustly simulates topographies of drinking in young adult humans [99]. Although there were no changes in frequencies of circulating white blood cells between the ethanol consuming and control animals, robust changes in gene expression were detected, suggestive of a robust effect of ethanol and its metabolites on regulation of gene expression within immune cells.

Among these gene expression changes, we saw a larger number of down-regulated (661 DEGs) compared to up-regulated genes (445 DEGS). Upon gene enrichment, a significant number of up-regulated genes mapped to blood coagulation. Notable up-regulated genes were involved in hemoglobin synthesis (*HBB*, *HBA2*, *HBG2*, *AHSP*, and *NFE2*), coagulation (*F13A1*, *F2R*, *ITGA1*, and *ITGB5*) and platelet adhesion (*SELP* and *GP5*). In contrast, genes known to inhibit coagulation (*SERPINB2* and *PLAT*) were down-regulated. In addition, our findings suggest an increased shift of the hematopoiesis transcriptional machinery towards erythroid lineage. This includes transcription factors involved in various stages of erythrocyte development (*GATA1*, *GATA2*, *KLF1*, *EPB42*, *TAL1*, *GFI1B*, and *MEIS1*) as well as hematopoiesis specific enzymes and regulators (*AHSP*, *ALAS2*, *ANK1* and *BPGM*). The imbalance in the expression of procoagulant, anticoagulant and fibrinolytic factors together with a shift towards erythrocyte development, suggests that chronic heavy ethanol consumption results in increased coagulation and an increased predisposition to cardiovascular diseases (CVD).

Indeed, additional functional enrichment analysis shows that up-regulated genes map to a number of CVD including anemia, myocardial ischemia, infarction and heart diseases. Recent studies have shown that *APOE* is a marker for CVD in women with high cholesterol and C-reactive protein levels but not in men [66]. Additionally *ALOX15* and *GPX1* were up-regulated in this study, which have been associated with higher risk of CVD [64, 65, 100–103]. These findings are consistent with clinical observations that heavy alcohol consumption is associated with alcoholic cardiomyopathy [104, 105]. Indeed, previous studies on the association of ethanol with hemolytic parameters such as the Framingham Offspring Study also reported that fibrinolytic potential decreases with increased ethanol consumption especially at doses greater than 7 drinks weekly and this could explain the increased risk of CVD in the drinking cohort [106]. Our observations are also in line with an increased incidence of thrombosis in beer-drinking men based on measurements of *PLAT* [107].

Heavy ethanol consumption interferes with several stages of wound healing including inflammation, angiogenesis, and restoration of extracellular matrix [108]. In support of these observations, several genes necessary for wound healing were down-regulated in our study including *CCL2* and *TGFBR1*, which are involved in angiogenesis and remodeling of extracellular matrix. Additionally, we also saw up-regulation of anti-angiogenic factors like *PF4*, *PF4V1*, *ANGPT1*, *SPARC*, and *SPARCL1*. Furthemore, plasma levels of VEGFD were significantly reduced (S1C Fig), a finding that agrees with previous studies on ethanol-consuming male cynomolgus monkeys [109]. Thus, the altered expression profiles detected in our study supports the clinical observation of delayed wound repair as a result of chronic ethanol exposure.

We also observed up-regulation of a large number of genes involved in immune signaling and inflammation (*BTK*, *CCL5*, *CCL4L1*, *CCL23*, *PF4V1*), interferon stimulating genes (*IFIT1*, *IFI44*, *IFI44L*, *IFIT1B*, *IFIT2*, *ISG12*, and *ISG15*) and activation markers (*CLEC1B*, *CLEC4G*, *CD69*). Collectively, these observations suggest a dysregulation of the innate immune response where nonspecific inflammatory responses maybe enhanced but pathogen-specific responses maybe dampened. A large number of these DEGs are expressed by monocytes and DCs suggesting that ethanol exerts its biggest impact on innate immunity. This is corroborated by additional bioinformatics analysis using the native expression profiles of these genes in purified population of peripheral blood cells reported in ImmGen database [110] (S3A Fig).

In support of studies reporting increased susceptibility to infection with AUD [111–113], we report down-regulation of a number of genes important for host defense such as cytokines (*IL1R1* and *TNFSF11*) [114], chemokines (*CCL2*), and receptors (*TGFBR1* and *TLR8*). In contrast, we observed significant down-regulation of *NFKB* associated genes in PBMC with heavy alcohol consumption (*TRAF3*, *RCOR2*, *JAG2*, *TNFSF11*, and *RELB*). This dysregulated inflammatory landscape could be due to increased bacterial translocation seen with heavy ethanol consumption [2] and/or exosome signaling [115]. Moreover, important transcription factors required for activating immune responses (*STAT3* and *RELB*) were down-regulated [116–118]. Interestingly, using the same model we have previously reported reduced levels of STAT3 mRNA and protein levels in PBMCs of a larger cohort of rhesus macaques. Additionally we have shown that these changes in mRNA and protein levels of STAT3 are mediated by increased expression of microRNAs miR-181 and miR-22 and resulted in ethanol dependent disruption of growth factors (VEGF, HGF) and inflammatory cytokine production (G-CSF) in PBMC following phorbol myristate acetate(PMA)/ ionomycin stimulation[15].

Our data support previously published reports of elevated levels of *SNCA* (Synuclein-Alpha, FC = 75.7) mRNA in the blood of rats, monkeys and humans with alcohol use disorder (AUD) [119–122]. Therefore, mRNA levels of *SNCA* may serve as a robust biomarker of AUD across species. Additionally, in accordance with a previous study on circulating plasma protein biomarkers associated with AUD in cynomolgus monkeys, we saw increased plasma levels of IL7 [109] (S1C Fig). Interestingly, in the PBMC, we saw down-regulation of *IL7* (FC = 2.2, FDR = 0.08). This contradiction could be explained by the differences in tissue-specific gene expression profiles since IL7 is primarily secreted by the stromal cells of the thymus and bone marrow and not by circulating PBMC.

Our analysis also provides some insight into mechanisms of altered gene expression with chronic heavy ethanol consumption. We report down-regulation of miRNA molecules as well as changes in expression of chromatin modifying enzymes–writers and erasers. Several studies have provided increased evidence that miRNAs play a role in ethanol withdrawal [114], the etiology of alcoholism [123] and hepatotoxicity [124]. However, very few studies have investigated the impact of ethanol consumption on miRNA expression within immune cells. It was interesting to note that all the differentially expressed miRNAs detected my RNA-Seq were down-regulated. Of particular importance, miR-27a, which was significantly down-regulated, can regulate the M2 macrophage polarization of monocytes through regulation of ERK signaling and by the secretion of IL10 [75]. *IL10* was up-regulated in our dataset, as were three predicted targets of this microRNA. Similarly, several targets of the down-regulated miR-24, (*NEFM* and *BNIP3L*) were up-regulated on heavy ethanol consumption.

We also report up-regulation of histone genes, but down-regulation of all histone modifiers (writers and erasers) including deacetylases (*SIRT1*, *HDAC9*), acetyl-transferases (*KAT6A*), methyltransferases (*KMT2C*), and demethylases (*KDM5A*, *KDM2B*, and *JARID2*). These results suggest that chronic heavy ethanol preferentially induces post-translational modifications on histone H3. Interestingly, brain chromatin remodeling of histone H3 and H4 have been attributed to facilitating ethanol withdrawal symptoms [125]. Furthermore, studies from alcohol exposed rat hepatocytes and livers of alcohol-fed rats indicate changes in acetylation of histone H3 [126, 127]. While it has been well established that epigenetic changes in histone H3 and H4 play a role in regulating inflammatory responses in immune cells following LPS stimulation [128], whether alcohol exposure affects epigenetic mechanisms to prolong expression of cytokines in immune cells is not yet known. Therefore, future studies will focus on uncovering mechanisms that regulate gene expression changes including methylation, expression changes in non-coding RNAs, and histone modifications in immune cells and how it correlates with gene expression changes at key inflammatory loci.

## Materials and Methods

### Animals and Sample Collection

This study was performed in strict accordance with the recommendations made in the Guide for Care and Use of Laboratory Animals of the National Institutes of Health, the Office of Animal Welfare and the United States Department of Agriculture. All animal work was approved by the ONPRC Institutional Animal Care and Use Committee. Nine female rhesus macaques (average age 4 years 2 months) were used in this study, with three animals serving as controls. We used schedule-induced polydipsia to establish self-administration of 4% (w/v) of ethanol in the remaining 6 female rhesus macaques. All animals were housed in quadrant cages (0.8 × 0.8 × 0.9 m) with constant temperature (20–22°C), humidity (65%) and a 11-h light cycle with visual, auditory and olfactory contact with other conspecifics. The barrier between monkeys housed side-by-side was removed for 2h/weekday and the monkeys shared the expanded housing cage. The animals had access to environmental enrichments like toys. For the purpose of this study, the animals were not sacrificed.

Briefly, monkeys were trained to operate a panel inserted as part of the sidewall in their housing cage and could obtain all fluids and food through this panel [14, 129]. Ethanol self-administration was established using an induction phase that lasted 4 months, with the dose of ethanol (0, 0.5, 1.0, 1.5 g/kg) increasing every 30^th^ session. Following the induction phase, monkeys were allowed an “open-access” availability to fluids (4% w/v ethanol and water, n = 6 or water, n = 3) for 22 hrs/day with food available in 3 meals/day. The open access condition occurred daily for over 12 months and parameters of the daily ethanol intakes were used in the data analysis. The blood ethanol concentration (BEC) was measured using gas head-space chromatography (Hewlett-Packard 5890 Series II, Avondale, PA, USA; equipped with a headspace auto-sampler, flame ionization detector, and a Hewlett-Packard 3392A integrator)[12]. For gene expression analysis, unanesthetized blood samples were drawn from all 9 animals after 12 months of ethanol self-administration. Differentials were obtained from whole blood using a complete blood count machine (Hemavet; Drew Scientific Group, Waterbury, CT) calibrated for rhesus blood. PBMC were isolated by centrifugation over histopaque (Sigma, St Louis, MO) as per the manufacturer’s protocol. The PBMC were cryopreserved in Fetalplex™ Animal Serum Complex (Gemini Bio-Products, West Sacramento, CA)/DMSO until they could be analyzed as a batch.

### Flow Cytometry

PBMC were surface-stained with antibodies against: (1) CD4 (eBioscience, San Diego, CA, USA), CD8β (Beckman Coulter, Brea, CA, USA), CD28 (BioLegend, San Diego, CA, USA) and CD95 (BioLegend) to delineate the naive (CD28^+^CD95^−^), central memory (CD28^+^CD95^+^) and effector memory (CD28^−^CD95^+^) T cell subsets; (2) CD20 (Beckman Coulter), IgD (Southern Biotech, Birmingham, AL, USA) and CD27 (BioLegend) to delineate naive (CD20^+^IgD^+^CD27^−^), marginal zone (MZ)-like (CD20^+^IgD^+^CD27^+^) and memory (CD20^+^IgD^−^CD27^+^) B cell subsets. (3) A second tube was stained with CD3 (BD Pharmingen, San Diego, CA, USA), CD20, HLA-DR (BioLegend), CD14 (BioLegend), CD123 (BioLegend) and CD11c (BioLegend) to delineate monocytes (CD3^−^CD20^−^CD14^+^HLA-DR^+^) and dendritic cells (DC, CD3^−^CD20^−^CD14^−^HLA-DR^+^). Dendritic cells were further defined into myeloid (mDC, CD123^−^CD11c^+^) and plasmacytoid (pDC, CD123^+^CD11c^−^). The samples were acquired using the LSRII instrument (Beckton Dickinson Company, San Jose, CA, USA) and data analyzed using FlowJo software (TreeStar, Ashland, OR, USA).

### Cytokine, Chemokine and Growth Factor Analysis

The circulating cytokines were measured in the plasma using nonhuman primate Cytokine/Chemokine/GF (eBioscience, San Diego CA) 37-plex panel that measures IFN**γ**, IFNα, TNFα, IL1RA, IL1b, IL2, IL4, IL5, IL6, IL7, IL8, IL10, IL12p70, IL13, IL15, IL17A, IL18, IP10, IL23, sCD40L, SCF, MCP1, MIP1α, MIP1β, MIG, Eotaxin, ITAC, BLC, SDF1α, VEGFA, VEGFD, GCSF, GMCSF, BDNF, FGF2, NGFß and PDGFBB.

### RNA Isolation and Library Preparation

Total RNA was isolated from PBMC using the mRNeasy kit (Qiagen, Valencia, CA), followed by polyA selection to enrich messenger (m)RNA. 1μg of polyA-enriched mRNA was fragmented followed by cDNA synthesis using random hexamers (New England Biolabs, Ipswich, MA). This was followed by end-repair, ligation of adapters and size selection using AMPure XP beads (Beckman Coulter Inc, Brea, CA) to isolate cDNA templates of 320 nucleotides, which were subsequently PCR amplified. Each library was indexed using a unique barcode for multiplexing and sequenced on the HiSeq2500 platform (Illumina, San Diego, CA) to yield single-end 100 bp sequences.

### Bioinformatics Analysis

RNA-Seq data analysis was done in R using Bioconductor packages and using open source command line tools. Quality reports for the raw reads were generated using FASTQC. The reads were then aligned to the *Macaca mulatta* genome from Ensembl using splice aware short read aligner suite Bowtie2/TopHat2 [130, 131]. The transcript counts per gene were then summarized using the *summarizeOverlaps* function, which counts reads that align to exonic regions only. Since the libraries were non-stranded in nature, the reads were counted in a non-strand specific manner. The final files of RNA-Seq read samples have been submitted to NCBI (SRP064925).

Differential gene expression analysis was performed using GLM method from edgeR package [132] in Bioconductor. Differentially expressed genes (DEGs) were defined as those with a fold change ≥ 2 and a false discovery rate (FDR) of ≤ 5%. Functional enrichment of these genes was done using MetaCore™ (GeneGo™, Thomson Reuters, NY) to identify clusters of genes mapping to specific biological pathways or disease associations. To identify potential targets of microRNAs, TargetScan database [133] was scanned with the list of DEGs for possible associations based on computational prediction with a context ratio of at least 95%. ImmGen database was profiled to identify possible sources of transcriptional changes within the mixed population of cells [110]. Additional functional enrichment on select gene groups was performed using InnateDB Gene Ontology [134] and Transcription Factor Binding Site (TFBS) tool. Gene networks were constructed using NetworkAnalyst [135] identifying enriched gene networks in input genes.

## Supporting Information

S1 Fig **Cell counts and cytokine, chemokine, growth factor measurements** (A) Cell counts from whole blood. (B) Percentage of major cell subsets within PBMC. (C) Average concentrations of cytokines and growth factors that differed between ethanol-consuming animals and controls.
(EPS)

S2 Fig Principal Component Analysis and clustering of samples used in this study. (A) Principal Component Analysis and (B) Bar graph depicting the 8 most significant Gene Ontology (GO) terms enriched among up-regulated differentially expressed genes (DEGs), and (D) the down-regulated DEGs. The bar represents the number of DEGs mapping to each GO term while the line graph represents negative log (FDR) of the enriched term.
(EPS)

S3 Fig ImmGen profile of genes mapped to ‘Immune System Process’. (A) Expression profile of genes mapping to ‘Immune System Process’ across several cell types as predicted by ImmGen’s MyGeneSet application. Genes are represented as individual rows while each column represents a study.
(EPS)

S4 Fig Network Analysis. (A) Network image of the DEG that map to uniquely to ‘Signaling’ and show direct interactions.
(EPS)
